# Performing performance: young aspiring athletes’ presentation of athletic identity

**DOI:** 10.3389/fspor.2024.1383559

**Published:** 2024-08-22

**Authors:** Anette Skilbred, Åse Strandbu, Sigmund Loland

**Affiliations:** ^1^ Department of Sport and Social Sciences, Norwegian School of Sport Sciences, Oslo, Norway; ^2^ Norwegian School of Sport Sciences, Child and Youth Sport Research Centre, Oslo, Norway

**Keywords:** youth athletes, athletic identity, elite sport, interactionist perspective, performance, goffman

## Abstract

Youths are in the process of figuring out answers to the question “who am I?” and young athletes are searching for athletic identity in interaction with their friends, teammates, coaches, and so on. This study explores athletes' presentations of athletic identity based on 24 interviews with ambitious young athletes attending upper secondary sport schools. Anchored in Goffman's theory of the self and the presentation of the self, as well as Markus and Nurius' concept of possible selves, the study views identity as socially constructed in interaction. Utilising this theoretical perspective alongside thematic analysis resulted in four themes that reveal characteristics that are deemed central in an athletic identity. First, the theme *I am a dutiful athlete* constitutes integral facets of the athletes' self-presentation as committed and diligent individuals. The interviews also bring to light variations in the athletes' attitudes and approaches towards these expectations and concepts. The remaining three themes: *I must be unique*, *We must be unique,* and *I must have fun*, illustrate how being a performing athlete extends beyond duties tied to training, resting, and eating. While the findings suggest the existence of certain dominant and desirable characteristics in an athletic identity, they also highlight variations in identities, emphasising negotiation and flexibility in handling the athlete role.

## Introduction

Young people are engaged in the journey of self-discovery, which revolves around the question of “Who am I?” (1). They delve into the personal realm of self-discovery while seeking a sense of belonging. For young athletes, these processes also include figuring out their athletic identity, and they do so by interacting with their friends, teammates, coaches, and so on (2).

In the field of developmental psychology, identity is perceived as a developmental task—most pressing during the adolescent years. It involves the process of shaping one's self and understanding what holds significance in one's life, ultimately contributing to a sense of coherence. Psychologist Erik Erikson, a pioneer in this field, presented a theory of human development stages. In the stage of adolescence, handling the sense of identity vs. identity confusion is the priority task, where youths are “primarily concerned with what they appear to be in the eyes of others as compared to what they feel they are” (3). Psychological approaches to identity emphasise the idea of personal identity as the qualities and traits that define a person as unique. However, Erikson also emphasised the psychosocial part of identity development, and the importance of the community in which individuals live (4).

In the sport context, and especially for young athletes, it is crucial to present and demonstrate to oneself and to others that they are among “the chosen ones” who have the potential to be best in the future. Although the athletes have goals for the present day, many goals are long-term, meaning that several choices are made to bear fruit sometime in the future.

This paper explores aspiring youth athletes' presentations of their athletic identity based on interviews with 24 youth athletes from private elite upper secondary sport schools (PEUSS) representing various sports. We ask the following question: How do the young athletes perceive and present an athletic identity? Moreover, what challenges and tensions are associated with manoeuvring the athletic identity?

### Previous research: athlete identity formation and presentation in sport

Athletes' identity formation and presentation have been explored from various perspectives. One tradition perceives athletic identity as “the degree of personal connection to sport” (5), which is measured with the Athletic Identity Measurement Scale (AIMS) (6). A review by Edison et al. (5) synthesised previous research in this tradition on how the degree of athletic identity correlates with various variables and revealed, amongst others, that athletes with a high degree of athletic identity encounter greater difficulties in navigating a career transition. In this same tradition, Brewer and Petitpas (7) reviewed qualitative and quantitative literature addressing “identity foreclosure”—a state of strongly committing to a role and not engaging in alternative roles and identities, which is found to often be the case for youth athletes as they use a lot of time and energy engaging in their sports. Brewer and Petitpas' review reveals conflicting results related to having a unidimensional identity. For example, a strong athlete identity is both positively and negatively associated with burnout. From another perspective, Ronkainen et al. (8) reviewed qualitative research that understands athletic identity as “constituted within cultural narratives and discourses available to the individual” (p. 135). This tradition addresses how cultural values and life scripts shape athletes' identities, the role of language and power dynamics in the identity construction and how the sport contexts are most often dominated by discourses favouring performance. This line of work challenges static conceptualisations of identity. Based on the review, Ronkainen et al. (8) conclude that the pool of identities available for athletes' is too narrow and inhibits athletes' identity construction as multidimensional humans, not just athletes. The complexity of athlete identity formation is further emphasised in Kavoura and Ryba's (2) study on how the athletes' construction of identity is intertwined with their management of dual careers. Kavoura and Ryba describe negotiation processes influenced by culturally dominant notions of “a good athlete” and explore how changes in sport and education policies shape young athletes' perceptions of their future. Their findings indicate that athletes may encounter identity tensions and reduce their athletic aspirations while striving to fulfil demands and expectations.

The work of Carless and Douglas is especially relevant as they view identity as a process where athletes perform or “present themselves” in the role that is commonly expected and fits with the script of *the performance narrative* (p. 706). The performance narrative implies that “performance-related concerns come to infuse all areas of life while other areas are diminished or relegated” (p. 702). How athletes relate to the performance narrative does however vary. Carless and Douglas' (9) identified three ways; The first is “living the part of the athlete”, where athletes conform to the narrative by prioritising routines and success over relationships. The second is “resisting the part of the athlete”, where athletes maintain a multidimensional identity by prioritising relationships outside of sport. The third identity is “playing the part of the athlete”, characterised by adapting routines based on context and audience, suggesting a multidimensional identity that is occasionally overshadowed by the performance narrative.

Ryba et al. (10) interviewed athletes in dual career positions, similar to our sample, examining how they construct their identities and future narratives. Generally, athletes predominantly drew on the performance narrative to craft their life stories, and some of the athletes struggled to envision a future beyond their athletic identities. This means the athletes to a strong degree construct and present their athletic identities in line with the performance plot where being best and training hard is central aspects. The authors suggest that the athletes' narratives about their future selves were notably sparse in providing detailed accounts of their non-athletic identities. However, by incorporating athletes' small stories into the life story approach, they demonstrated how athletes can narrate an athletic performance story while including other aspects of life and self.

In our study, we view athletic identity as a cultural construct shaped by societal expectations and values regarding athleticism within a specific context and align with previous research, seeing identities as “fluid and multiple (rather than stable and singular), and acknowledge that identities are performed in a social context and cannot be assessed in isolation from the cultural context” (8).

Our research draws inspiration from the literature that explores the constructive elements of athletic identity, examining how athletes present what we interpret as preferred athletic identity characteristics within their sporting contexts. Our research contributes to filling the gaps by exploring presentation of athletic identity among young athletes in secondary sport schools. This group of athletes is growing, in line with the increasing number of schools (11) and has been relatively well-researched over the past decade (12–14). However, presentations of athletic identity in relation to a performance perspective, which has received more attention in other Nordic countries (2), remain unexplored in the Norwegian sport school context.

## Youth athletes' presentations of self: an interactionist perspective

Identity is one of the most explored constructs in social science (15). Sociologically inspired research on identity underlines identity as cocreated in interaction with other people and dependent on the context (16). According to Jenkins (16), all identities are social by definition: “Identifying ourselves or others is a matter of meaning, and meaning always involves interaction: agreement and disagreement, convention and innovation, communication and negotiation” (p. 4). Jenkins’ (16) quote highlights the social interactional part of the meaning-making of identity: “We work at presenting ourselves so that others will work out who we are along the lines that we wish them to” (p. 6).

Goffman's (17) dramaturgical approach provides a valuable framework for examining the presentation of athletic identity among young athletes, as also discussed in Jenkins' book *Social Identity* (16). Goffman conceptualises life as a stage where individuals perform different roles, distinguishing between the “frontstage” and “backstage” areas of life. The frontstage represents the public realm where individuals actively manage their image and align their behaviour with how they wish to be perceived by others. The backstage, however, is a space where individuals can lower their guard and engage in less staged behaviours, potentially revealing contradictions and a more relaxed presentation of themselves that is not intended for public view.

For young athletes, the frontstage is where they consciously project their athletic identity to peers, coaches, and the public, striving to meet the expectations and norms associated with their sport. This involves strategic impression management to align with their desired athletic role. The backstage is where athletes might show different aspects of their identity and express feelings that are less polished and more varied. Goffman's (17) concept of impression management is crucial here; it highlights how athletes continuously craft and negotiate their identities in response to social pressures and expectations.

Jenkins (16) supports this view by discussing the dynamic interaction between self-image and public image. According to Goffman, the self is not a fixed entity but rather a collection of roles or “masks” individuals present in various situations. This perspective helps to understand how young athletes actively construct and maintain their athletic identity through their performances and interactions.

Overall, self-presentations and impression management take place in what Goffman calls the interaction order, which he defines as the shared rules and expectations that individuals in the same context use to coordinate their daily sense-making and social relations. This order is created in micro-sociological interactions and arises in spite of authorities and not because of them. The order makes conventions possible, like rules in a game (18). Goffman's (17) work is the foundation for the understanding of the self in our study, or, in our terminology, for the understanding of identity.

In addition to the presentation of the self, Goffman (17) provides perspectives on how individuals coordinate team presentations. He uses the team concept to illustrate the work of a group of individuals who cooperate in a performance, attempting to achieve goals for the group's sake. Goffman explores the group dynamic and the relationship between performance and audience. In our study, we interpret the pronoun *we* in the interview as an expression of a team performance. Since the athletes shift between using “I” and “we” in their stories, we find it relevant to explore identities on several levels.

According to Goffman (17), individuals tend to conceal or underplay the activities, facts and motives that are incompatible with the idealised self-version when they present their routines. We, therefore, assume that athletes are likely to present routines and goals associated with athletic identity ideals that are considered meaningful in their context. However, the methodological conditions, such as anonymity, may also facilitate backstage presentations, which will be more elaborated in the method section.

Goffman's work has been criticised for representing a narrow Anglo-American view of the self and for lacking the perspective to explore how individuals navigate multiple alternative identities and the multidimensional nature of identity (16). Markus and Nurius's (19) theory of possible selves provides a thorough response to this critique by examining how identities in the present are shaped by future-oriented self-concepts. Their concept of possible selves refers to the various identities individuals envision for themselves in the future, including both desired and feared outcomes. This framework offers valuable insights into how future goals and aspirations influence current behaviour and motivation, thereby enriching our understanding of identity formation and management beyond the scope of Goffman's original theory (20).

Markus and Nurius describe possible selves as a type of self-knowledge that “pertains to how individuals think about their potential and about their future” (p. 954). Although these possible selves are future-oriented, they are closely linked to the current self, which they term the working self-concept.

According to Markus and Nurius, these selves are both personal and social, often shaped by past social comparisons. They explain this with the following quote:

An individual is free to create any variety of possible selves, yet the pool of possible selves derives from the categories made salient by the individual's particular sociocultural and historical context and from the models, images, and symbols provided by the media and by the individual's immediate social experiences. (19).

This indicates that possible selves are influenced by the sociocultural forces present in the context. In our study, the young athletes are situated within a high-achievement context, and the athletic identities they present can offer valuable insights into how identities are manoeuvred and relates to validation of certain identities. Additionally, the goal-setting habit in sports inherently directs young athletes to focus on their possible future selves.

Goffman's work and Markus and Nurius' work is used complementarily in the analysis presented in this paper. Goffman's framework is predominantly used to address the first research question: How do the young athletes perceive and present an athletic identity? This approach explores the presentation and performance of athletic identities in the social context, enabling presentation of both front and backstage. Markus and Nurius' concept of possible selves is especially relevant for addressing the second research question: What challenges and tensions are associated with manoeuvring the athletic identity? This perspective provides a lens for understanding how athletes manoeuvre their athletic identities, especially current self-concept with their envisioned future selves.

## Method

In this section, we describe philosophical premises, some of the athletes' shared characteristics, the context, the interview research process, recruitment, interviewing, analysis, methodological reflections and rigour, and ethical considerations.

### Philosophical premises and authors positioning

Our interpretation and application of interactionist theory is based on the constructivist paradigm. This means that we consider social reality as constructed, and that the athletes meanings about the topics of interest are constructed in interaction among them and with other actors in their context, including the first author conducting the interviews (21). The first author, who conducted the interviews and performed the main part of the analysis, had no prior affiliation with the schools involved in the study. The first author's limited insider knowledge regarding the schools, allows for a more naïve approach to the research, facilitating an exploration of the data with fewer preconceived notions or assumptions about the institutions. However, due to the public debates surrounding these schools, the first author is not entirely devoid of contextual information. The author groups' sole connection to the institutions is through their research activities.

### Context and population

The only criteria for joining the study was that athletes had to attend a PEUSS. The first author contacted the administrative staff at three PEUSS. All three schools agreed to attend, and the staff forwarded the study information to athletes. Contact was established between the athletes who wanted to join the study and the first author. The athletes reviewed the informed consent form and were further informed about the study and their rights. The time and place for interviews were based on what was convenient for the athletes. The recruitment processes were characterised by self-selection among schools and athletes, as well as practical considerations like athletes' teaching and training and competition schedules. Some athletes had to withdraw for various reasons.

PEUSS, while not officially part of the Norwegian sports model, are recognised as crucial contributors to elite sports development. These institutions aim to make a significant impact on both elite athletics and society at large. With a distinct strategy and vision, they position themselves as educational alternatives that emphasise sporting and developmental opportunities for young, aspiring athletes. Their educational programmes are flexible, adjusting throughout the year to align with the demands of training and competition schedules. Additionally, these schools seek to benefit their local communities by sharing the expertise of their highly qualified coaches with local sports teams, collaborating on events and competitions, and providing access to their sports facilities (22).

The sample consists of twenty-four athletes aged 17 to 19 from three different PEUSS. The athletes practised various sports: ice hockey, handball, biathlon, motocross, track and field, swimming, cross country, and football. The majority had moved from their childhood homes to attend a specific school to develop in their sport. Many of the athletes expressed high ambitions and sacrifices in order to establish an everyday life that revolved around sport. Sport occupied most of their time, and they usually spent time with the same people in school, in sport and in their spare time. The athletes were concurrently balancing their involvement in sports careers and completing upper secondary school education. This was evident through multiple statements indicating a scarcity of time for activities beyond school and sports.

### Interview and analysis

Initially, interviews were planned as one of several empirical inputs, among them observation. However, due to COVID-19, observations were cancelled, leaving only interviews as the means to engage athletes in discussions about their interactions with others and their presentation of themselves in the sport context. In most cases, interviews were held at the school, with only two conducted via video. All the names used in this article are pseudonyms.

Interviews lasted from 40 to 70 min and were semi-structured, warranting the same topics were covered in the interviews with all athletes. The interview guide contained questions such as: “what do you think it takes to be the best in your sport?”, “what do you think people at the top of your sport have done to get there?”, and “are there any supplements that are visible in your sport?”. The aim was to enable accounts that could facilitate an analysis of preferred characteristics in the athletes’ presentations and get insights into their reflections related to performing in their sport.

In our analysis we were especially interested in the expressions with a self-presentation form (17). Furthermore, we aimed to identify nuances and tensions athletes face and manoeuvre arising from expectations placed upon them. In addition to being grounded in the interactionist perspective, our analysis utilised Braun and Clarkes' (23) reflexive thematic analysis with its systematisation and phases for examination of interview material. The initial analysis phases were conducted by the first author and included the transcription, reading, coding and the first construction of themes. When revisiting the themes, the author group discussed the themes in terms of characteristics and quality.

Practically, the analysis followed a systematisation in phases, starting with searching for quotes concerning athletes’ descriptions of athletic identity and quotes relating to athletes' reasoning and ideas related to being able to perform well (9). Additionally, considering our assumptions that the front- and backstage will emerge and shift in the interview, quotes related to a negotiation of the correct way to conduct oneself as an athlete and present the athlete identity were extracted. When all relevant quotes were extracted, the next step involved labelling a meaningful word or short sentence that represented the meaning in the quotes. This coding process resulted in 101 codes. Some quotes have several codes, illustrating the negotiation between various ideals and expectations in athletes' presentations. Afterwards, codes that shared descriptive characteristics were grouped together, facilitating the systematic organisation and analysis of related content. This means that descriptions of duties related to, for example, training and resting were clustered together, as well as descriptions of their striving to stand out, allowing for a more comprehensive understanding of the various aspects of their roles. Before reaching the final four themes, as outlined here, the themes underwent multiple discussions and revisions (24). During this phase, the codes remained unchanged; however, they were categorised in various ways in an attempt to grasp certain aspects in athletes' presentation of their athletic identity.

### Methodological reflections and rigour

Furthermore, when it comes to the conversations about ideal and future identities, the focus was less on immediate actions and more on providing the athletes with a chance to contemplate their roles, including aspects related to their behind-the-scenes experiences. Some central reflections need to be addressed. First, the frontstage and backstage pending, second, whether we are capturing the “social” nature of athletic identity and third, if the ideal of “athletic identity” can be distinguished from the individual confrontation and concrete manoeuvring of this ideal.

Grounding our study in the interactionist perspective (17), we propose that athletes' self-presentations in interviews reveal some idealised athletic identity qualities in context-specific ways. We see interviews as frontstage settings where athletes align their narratives with accepted norms for how athletes should present themselves. However, the interview context—characterised by anonymity and its duration—may also encourage athletes to share more nuanced, backstage aspects of their experiences, including identity adaptations and paradoxes. We suggest that athletes navigate between frontstage and backstage presentations during interviews. For instance, when discussing performance enhancement with a researcher from the Norwegian School of Sport Sciences, athletes may present a polished frontstage image. Simultaneously, the impression from the interviews was that the anonymity of the interview allowed for more candid backstage disclosures. This dual presentation offers valuable insights into athletes' lives and meaning-making, aligning with Goffman's view that both stages involve staging.

Considering the use of interviews to obtain athletes' accounts of their athletic identity and the construction of identity through social interactions, it is worth discussing whether this fully captures the “social” nature of identity. Observations might have provided more immediate insights into how athletes construct and present their athletic identities through their interactions with others in their everyday life context. However, interviews enable athletes to reveal qualities of their athletic identity that are not easily observed. By utilising Goffman's work on the presentation of the self and including questions about social interactions, we argue that our interviews capture some of the social qualities in athletes' presentation of their athletic identity.

The final point that warrants reflection is the relationship between the ideal of “athletic identity” and the way individuals manage or navigate this ideal in their personal experiences. The former refers to normative expectations and ideals regarding what constitutes the perfect athlete. It encompasses the characteristics, behaviours, and attributes that are considered ideal or aspirational for athletes. The latter involves how individual athletes engage with, adapt to, and navigate these societal ideals. This includes their strategies for aligning with or responding to these expectations, as well as the ways they manage the pressures and challenges associated with the ideal athletic identity. Analysing differences between societal ideals and the individual management of these ideals is not the primary focus of this paper. In line with Goffman's perspective—how masks, roles, and selves come into being during interaction (25)—we argue that both aspects are equally relevant in presenting athletic identity.

### Ethical considerations

The project has been approved by the Norwegian Centre for Research Data (NSD), and all athletes signed the informed consent before the interviews. To ensure athletes' confidentiality and anonymity, we use pseudonyms that only reveal the participant's gender. The interviews were conducted in Norwegian, and we translated them with caution when reporting the citations. When needed, we have edited the quotations carefully to ensure the meaning is retained (26).

## Findings

This article aims to explore the characteristics of athletic identity by examining athletes' presentations of their habits, actions, and goals. The first theme is named, *I am a dutiful athlete* and conveys topics related to training, rest and nutrition. These duties tend to dominate young athletes’ sporting lives as obedient athletes.

The last three themes, *I must be unique, We must be unique*, and *I must have fun,* highlight different strategies in athletes' presentation of their athletic identity. These themes reveal how athletes navigate and express their individuality and collective distinctiveness, as well as the importance of enjoyment in their sporting lives.

### I am a dutiful athlete

The athletes describe a dutiful youth athlete as someone who embodies commitment, discipline, and respect both on and off the field. The dutiful athlete prioritises his or her sport, adhering to training schedules while also ensuring proper rest and recovery, and understands the significance of nutrition. The dutiful self is illustrated through training, resting, and nutrition, but also permeate into other choices in their daily lives. This is demonstrated in the athletes use of the “24/7 athlete” term, which seems to have an impact on how the athletes consider the “right” choices in line with their athletic identity.

Robert explains:

*When it comes to being what they call a 24-hour athlete, it's like that you eat enough, and you eat correctly. Of course, you're allowed to enjoy yourself, but get what you need, right. And that you train with quality, at least if you have a vision of progressing.*

Robert's quote illustrates a tendency in several of the athletes’ accounts: No single factor ensures good performance. Athletes emphasise the importance of making multiple correct choices to succeed, which requires prioritising. Emma's quote illustrates how the prioritising can look like:

*There are a lot of things that you prioritise, so maybe some things you choose to leave out and that you have to go home early if you are with friends and go home to go to bed early to get enough sleep and that you eat enough and that you recover enough and there is a lot that you have to be a bit egoistic about.*

Athletes' dedication to their identity is evident in their training. In interviews, they emphasise that training with a serious attitude is significant. They demonstrate significant self-discipline and willpower. They stress the importance of being well-prepared, focused, and hardworking, concentrating on their weaknesses. In other words, it is not enough to merely show up; serious athletes actively train themselves. Olivia says:

*You must have the right attitude towards training, you have to work for things, like you don't show up for training to be trained, but to train.*

Jack expresses the importance of sticking to the plan and going to training sessions even though you may be tired:

*Sometimes you feel shit when it's a normal training, and think maybe you should skip the training session and you're really tired. But there are also in those hardships, when you have bad days that if you manage to readjust your head and sort of think “just get through it, then I’m done”. It's in the hardships that you really get better.*

When faced with a heavy training regimen, rest becomes crucial. Sleep and rest are as important as training for reaping its benefits. Athletes describe rest as intentional recovery, skipping sessions, or power napping between school and training. They often express that rest is as important, if not more so, than training. The value of training is realised through adequate rest, as illustrated by Christina:

*I want to be involved in as much as possible all the time and then it becomes very much like, that it becomes very quantity over quality. So lately I've had strong focus on sleeping, preferably nine hours if possible, otherwise I don't function.*

Elisabeth describe how she got more aware of the balance between training and resting after one in her team got an overuse injury:

*Maybe I need to be a bit more mindful about taking rest days and ensuring enough recovery and food intake, and, you know, not just training all the time. I need to take it easy and so on because it's not something I've thought much about before. I'm not used to training so much, and I've never really considered that one might not be getting enough recovery, in a way.*

Sleep and rest are also emphasised as a priority concerning long-term goals, as youth athletes plan for peak performances in the distant future. Ella's quote emphasises that to reach the long-term goals, one must see the big picture:

*Recovery that's really the most important thing. It's not right now we should be good in a way. We shouldn't be afraid to take the extra day of rest because, in the big picture, it doesn’t matter if you trained that day. Because it's all in total, not that day in a way, that makes you good.*

In this big picture, the athletes, unsurprisingly, are concerned and thoughtful about food and eating. They regularly learn about nutrition at school, and they are encouraged to handle food in a healthy way.

Whether it is healthy or unhealthy, the bottom line is that a dutiful athletes get enough food that balances their activity level. However, the athletes describe it as challenging living up to the ideal of planning meals, making the food, and ensuring the right nutritional components are in each meal throughout the day. Ella's quote illustrates the challenge:

*First of all, you have to manage to bring enough food. Some days it can just be like that oh shit today I have too little with me because I had another training session which made me more hungry.*

The athletes are disciplined in their eating habits, following a structured meal plan with desired components consumed quickly after training. However, they also expressed different perspectives, such as eating unhealthy food to compensate for high activity levels and ensure sufficient energy. Ella's quote shows that while sweets are acceptable, healthy food remains a priority.

*Most people are like that, they eat a lot of sweets but eat healthy food first. And that's in a way how we're trained to, that somehow, if we eat proper food first, then we can eat sweets.*

This shows the disciplined nature of athletes while also indicating a non-obsessive relationship with food. Although athletes vary in how they present meals and approach nutrition, they agree on aspects like meal timing, reflecting prevailing norms.

In sum, athletes' dutifulness encompasses several aspects and extends beyond training, resting, and eating. Athletes face diverse choices to demonstrate their dedication and seriousness.

### I must be unique

Athletes' accounts demonstrate a quest for uniqueness, emphasising willingness and the importance of standing out in the group. The athletes describe various strategies in order to give the impression that “I am somewhat unique”. It can take the form of being better prepared for training, showing up earlier for training, excelling at handling food, and training more or smarter than others.

One tendency when it comes to being unique is the idea that one should accumulate and escalate what others do in order to distinguish oneself. To distinguish themselves in the relatively homogeneous group, the athletes express the necessity to incorporate something or exhibit a trait that others do not possess. Alexander states:

*It's about being focused on the small details that no one else does and that you are, in a way, a little independent compared to the others. The others may exercise exactly the same, but then you do the extra little things that need to be done. It may not necessarily be that you exercise the most and go hardest in the sessions, but that you train the smartest.*

It can also mean putting in more training, as Robert describes:

*Push hard. Also, I tend to often stay 10 min to a quarter after the session to practice some individual things. I feel that has helped a lot. And it is sort of, what should I say, a bit like make or break on who wants to continue and not.*

This quest for uniqueness seems to be a way of giving meaning to their everyday life that is strongly influenced by being an ambitious athlete. Presenting themselves as unique becomes particularly important as they surround themselves with other athletes who are just as ambitious. The desire to stand out and be someone who “goes the extra mile” is probably also a more or less deliberate attempt at self-conviction, and for holding on to the belief that “I will be the best athlete in the future”.

### We must be unique

In addition to the presentations of individual exceptionality, there are several examples where the athletes express a collective uniqueness. So, in addition to be an individual with a desire to stand out and put in the extra effort, there is also an ongoing group identity presentation. When the athletes use *we*, they usually refer to the sporting community in which they practice, and the way it is expressed bears some similarities to the ideas of individual uniqueness. Many times, it involves presenting one's own sport as somewhat more favourable compared to others.

One example of a collective expression of uniqueness is illustrated by Helena when she describes some differences in seriousness:

*I think we are quite similar in the class because we are put together [my sport], [sport] and [sport]. So, it's pretty much the same type of sport, but across the sports like that if you compare [my sport] then vs. hockey or handball, I would say that we are perhaps a bit more serious. Or it requires more from us than it perhaps does from them to achieve the same then, but it is interesting to see how the other sports also solve it.*

Another example is found in Luca's quote:

*I think [my sport], has sort of always been the sport that has trained the most. We have a fitness room in the [arena], we have [track]. We kind of have everything, so it's much easier to put in more training.*

Luca does not explicitly compare to another sport but still expresses that his sport possesses some desirable characteristics. Alexander's quote includes a comparison and an emphasis that in his sport, there is more focus on functionality rather than aesthetics:

*There is no [appearance] pressure in [my sport]. More so in other sports such as football and such, where I think they care more about things like that. In a way, we want to have the best possible function in the sport. We don't want to look the best, but go a little slower. We want to be the best possible version of ourselves.*

In sum, this theme illustrates a collective identity that highlights the sport group's uniqueness. This sense of uniqueness contributes to the group's overall cohesion and helps to reinforce their shared values.

### I must have fun

*I must have fun* is described as an important quality by many of the athletes. Across gender and sport types, all athletes accentuate that there must be a certain level of fun, motivation, or joy in their involvement. This desire for fun is not limited to the joy of performing well but relates to the pure joy of the unfolding of the sport itself. Fun is described in autotelic ways: as a value in and for itself. The athletes emphasise that you must continue to have fun and enjoy the sport to be able to endure a sporting career. Having fun along the way seems to be very important, as the sport takes up a lot of their time.

Victoria explains why fun is important when exerting much time on sport:

*I actually think that most athletes are quite strange because it is quite strange to spend so much of their time on one thing and they sacrifice alot. You have to sacrifice a lot but as I said previously, I think it's very important to have fun in order to, sort of, preserve that spark with what you do.*

They describe the fun of doing sport as more valuable than other forms of distractions, such as having fun at a party. Lucas explains:

*But the thing is that it's somehow not tempting [to go to a party] because if I do it then I kind of know the consequences of what it destroys for [sport], and I’d rather have more fun with developing myself and become a better [sport] player than to have fun at a party like that. For me it's about prioritising what you think is fun, is it partying or is it playing [sport]?*

Also, fun is highlighted as a desired factor when describing their role models in sport. Tomas describes how one of the best athletes in his sport from Norway has retained the playfulness and joy in his practice of elite sport:

*I think he has worked, he hasn’t given up, he has worked very hard. But at the same time, he has said himself that he has had a lot of fun the whole time. He is a very playful type, you can see on [track]. And he has that kind of skill and playfulness with him all the time.*

It is worthwhile noting that athletes’ descriptions of the importance of having fun and feeling joy in sport are often accompanied by references to what appears as the contrary: seriousness. Expressions such as “do not give up”, “hang in there”, and “grit your teeth”, occur several times alongside the message that sport must be fun.

Emma says:

*I think it is very important to enjoy sports and that you manage to keep up motivation and you think it's fun. Because I think that once it's not fun, it just becomes so tiring to keep doing, it's like, you have training sessions morning and evening every day and you have to have something to keep you going, if not then I don't think you will be able to last long.*

In sum, our overall impression is that the athletes experience much fun and enjoy life as youth athletes. However, when they combine the need for fun to persevere in the sport and the high training load, this also appears as a way of justifying and giving meaning to their distinct lifestyle.

## Discussion

The four themes presented in the findings section are key characteristics and qualities in athletes' presentations of their athletic identity, illustrating how living in a performance-driven context shapes their sense of self.

In this discussion section, we will pay particular attention to the nuances and tensions within these four themes, which we interpret as expressions of how athletes navigate their athletic identity. We will discuss the themes separately, and in relation to each other, relate the themes to the theoretical framework and suggest how the findings expand the field.

The first theme, *I am a dutiful athlete*, comprise some dominant discourses and narratives seen in previous research on athletic identity. In this theme, a performance logic is evident through the athletes' descriptions of —training, resting, nutrition—and also how dutifulness is demonstrated beyond these topics. Athletes' dutifulness is further exemplified through choices related to lifestyle, social engagements, and personal sacrifices. This commitment is particularly evident in how athletes approach their training sessions. There is a strong consensus that athletes must approach their training sessions with the “right” attitude and presence, thereby showcasing their discipline and commitment.

The difficulties of balancing training and rest are evident in several of the athletes' accounts. Although they may not explicitly frame it as a balancing act, they recognise rest as a crucial element when aiming for long-term goals. Athletes may justify skipping a training session by emphasising that the focus should be on peak performance in the future rather than immediate results. This perspective reflects a futurisation strategy, where the current self is oriented towards constructing future possible selves (19), thereby shaping their actions and decisions based on long-term aspirations. The central challenge appears to be the ability to internalise and maintain this long-term perspective.

When it comes to food and nutrition, similar concerns emerge. Many athletes adhere to guidelines taught by their educators, such as following the “plate model,” consuming fish a certain number of days each week, and limiting energy drinks (27). Conversely, athletes also describe their relationship with food as one that includes enjoying sweets and indulging in less healthy options, all while striving to maintain a “normal” relationship with their diet. Often, the primary concern for athletes is simply to ensure they consume enough food, whether “it is a burger or a salad with chicken”, to use Erik's own words.

In the second theme, *I must be unique*, athletes articulate the need to distinguish themselves within their group. As highlighted in the findings, this drive for uniqueness can manifest in various ways, such as arriving at training earlier, staying later, being more meticulous with their diet, or prioritising additional rest and recovery compared to others. The literature presents differing views on the need for distinctiveness: some theories suggest that cultures with values emphasising uniqueness foster distinctive identities, while others argue that the quest for uniqueness is a fundamental human need (28). The athletes’ pursuit of uniqueness often drives them to adopt strategies that set them apart from their peers, which not only influences their training routines but also reinforces their sense of individual identity within the group. This pursuit aligns with the view that the need for uniqueness can be a powerful motivator in sport contexts, supporting the idea that personal and cultural values around uniqueness play a significant role in athletes' presentations of their athletic identity.

Interestingly, this study also reveals a quest for group uniqueness in theme three, *We must be unique*. The athletes not only seek to stand out individually but also aim to be part of a sport that distinguishes itself from others. This finding resonates with Goffman's (17) descriptions of self-presentation and team performance, which emphasise the identity process as involving both individual and group validation. The athletes in our study demonstrate their group membership by highlighting the unique qualities of their sport group. They emphasise both their group's distinctiveness and their own personal uniqueness.

Theme four, *I must have fun*, highlights the importance of enjoying the sport and maintaining an element of playfulness in their approach. The fun described by the young athletes carries significant meaning. They articulate their need for fun as a blend of playfulness and passion, resonating with classic theories of the joy of play, such as Johan Huizinga's *Homo Ludens* (29). According to Huizinga, play is a rule-defined, autotelic activity that requires players to engage fully and completely, embodying an all-encompassing attitude of “non-serious seriousness.” The young athletes' references to fun and play likely reflect both their socialisation into a sporting culture that values fun as a component of success and their personal experiences of meaningful engagement in their sport. This reference is not to the “superficial” fun of a party, but to the process of playing as a meaningful, even existential, activity that reveals something about their identity. The athletes present not only positive possible selves (19) who envision a fun future in the sport but also their current selves that experience fun in their everyday activities. Many of the athletes express that the presence of fun is crucial for envisioning a future in the sport; without it, they do not see themselves continuing in their athletic pursuits.

In summary, all four themes coexist as both topics and strategies in athletes' presentations of their athletic identity. However, while these characteristics appear to align, there is notable opposition between them, and athletes must navigate these conflicting aspects through negotiation. This dynamic interplay suggests that the presentation of athletic identity is not a straightforward process but involves managing complex and sometimes competing elements that influence how athletes perceive and communicate their identities.

First, the dutiful theme and the fun theme represent different values in the athletic identity. Athletes often discuss how they balance these aspects, highlighting the importance of both fun and perseverance in their success. This balance can be seen in two ways: as a contradiction, where having fun and staying dutiful might seem at odds, or as complementary features that together define the athletic identity. Athletes may view enjoyment and being dutiful not as mutually exclusive but as intertwined elements that collectively enhance their overall performance and commitment to the sport. We argue that this is a fine line, as athletes often combine these aspects when explaining what they believe is crucial for excelling in their sport.

Second, the “I” vs. “We must be unique” theme represents a negotiation for the athletes. In real-world, the pursuit of uniqueness for both the individual athlete and the group can pose challenges, particularly when the goal is to achieve distinctiveness as both a top athlete and a member of a leading team. This suggests that two powerful forces—one driving individual uniqueness and the other reinforcing group identity—can operate simultaneously. For young athletes, who may have less capacity to recognise when to slow down, both demands for uniqueness can be challenging and potentially overwhelming. The drive to assert the sport group's uniqueness, especially in comparison to other sports, can intensify the pressure to not only excel personally but also to elevate the group's status.

Regarding theoretical contributions, our interpretations align with both Goffman's and Markus and Nurius' perspectives. Athletes' presentations of preferred qualities on the frontstage and their backstage manoeuvring, combined with the futurisation aspect emphasised by Markus and Nurius, shed light on a core practice in sport: setting long-term goals to achieve one's aspirations.

In other words, athletes' “working self-concept” is significantly shaped by their future possible selves, as illustrated by their approach to balancing rest and training. We interpret this as follows: while their current athletic identity motivates them to train as much as possible to build stamina, their decisions about whether to train or rest are influenced by their envisioned future selves. Ultimately, these future selves shape their choices and, consequently, their perspective on the preferred qualities in athletic identity. A true athlete understands the crucial balance between training and rest and manages the long-term perspectives necessary during their youth sport years.

Distinguishing between athletes' frontstage performances and alignment with societal expectations vs. their possible selves can be challenging. This means it can be difficult to determine whether their frontstage behaviour aligns with societal norms or reflects a manifestation of their individual possible selves. Consequently, their frontstage actions may not only be about managing impressions but also about aligning with their envisioned future self-concepts.

Because of the ethical considerations to anonymity, and therefore not revealing what sports are connected to the quotes or the overall findings, we cannot reveal and discuss individual differences, sport- specific differences and gender differences. This means that the nuances that are expressed in a quote from an athlete, can both be an individual manoeuvring, but also a manoeuvring related to a sport-specific cultural context, or gendered ideas related to the athletic identity. This would be interesting to explore in future research.

### Concluding comments

The goal of this article is to contribute to the study of the youth sport field by presenting athletes' perspectives on athletic identity. Combining theoretical tools from Goffman with Markus and Nurius enabled us to describe and discuss several details and aspects of athletes' identity work. This knowledge follows a line of research that has focused on the presentation of the self as more than *one* fixed identity (20, 30).

This study's exploration of athletic identity construction among young athletes expands the research field by addressing the multifaceted nature of identity formation in the sporting context. Furthermore, the study's emphasis on the interactional nature of identity construction underscores the importance of considering social dynamics and interpersonal relationships in shaping athletic identity. This highlights the need for future research to examine how peer interactions, coach-athlete relationships, and team dynamics influence the formation and expression of athletic identity among young athletes.

As in other research based on interviews, a methodological and epistemological question is whether the athletes' actions align with their accounts. We have no reason to doubt that the athletes' presentations might differ from their “actual” thoughts or conduct. Moreover, the discussions of ideal and future identities were less concerned with concrete actions here and now as they opened the opportunity for the athletes to reflect upon their roles, also relating to their backstage. The athletes did not only share their ideals when it came to handle everyday life, but also what they perceived as challenging and what they needed to mobilise to come as close as possible to the ideal athletic identity. These insights are of theoretical interest but also of practical significance to PEUSS coaches and staff. If athletes' ideals are unrealistic in relation to their present condition or predilections, it will most likely create a dissonance that can be hard to handle (31).

This research highlights the intricacies of identity formation in young athletes, a period marked by significant self-discovery and social interactions. By understanding how young athletes navigate their athletic identities within various social contexts, the study contributes to a broader understanding of their experiences and challenges. Exploring themes such as commitment, uniqueness, camaraderie, and enjoyment uncovers the diverse experiences and expectations that shape athletes' identities, offering a comprehensive view of their developmental journey.

Building on these findings, this research offers practical insights that can inform coaching strategies, youth sports programs, and support systems aimed at fostering positive athletic identities and holistic development among young athletes. For coaches, this means incorporating activities that balance fun with discipline, thereby encouraging both enjoyment and perseverance. Additionally, youth sports environments can benefit from creating settings that nurture playfulness and passion while also instilling a strong work ethic.

The understanding gained from this study is valuable not only for coaches, who can tailor training to enhance both individual and team development, but also for educators, who can integrate lessons on balancing sports with academics. Furthermore, the insights are pertinent for various individuals in the athletes' support network; sports psychologists can develop targeted interventions to support athletes' mental health and well-being, while parents can foster a healthy balance between sports and other aspects of life. Policymakers can use these insights to design youth sports programs that emphasise both competitive success and personal growth.

## Data Availability

The datasets presented in this article are not readily available because the transcribed interviews is only to be viewed by the first author. Requests to access the datasets should be directed to anettesk@nih.no.
