# Erratum: Performing performance: young aspiring athletes’ presentation of athletic identity

**DOI:** 10.3389/fspor.2024.1528022

**Published:** 2024-11-27

**Authors:** Frontiers Production Office

**Affiliations:** Frontiers Media SA, Lausanne, Switzerland

**Keywords:** youth athletes, athletic identity, elite sport, interactionist perspective, performance, goffman

An Erratum on Performing performance: young aspiring athletes’ presentation of athletic identity By Skilbred A, Strandbu Å and Loland S (2024). Front. Sports Act. Living. 6:1383559. doi: 10.3389/fspor.2024.1383559

Due to a production error, author Anette Skilbred was erroneously assigned as equal contributing author and author Sigmund Loland was erroneously omitted as equal contributing author.

The publisher apologizes for this mistake.

The original version of this article has been updated.

